# Impact of amino acids and sugars after thermal processing on acrylamide formation in synthetic potato models and real potatoes

**DOI:** 10.1002/fsn3.3818

**Published:** 2023-11-15

**Authors:** Nivine Bachir, Hadiya Akkoum, Montserrat Pujola, Franscesc Sepulcre, Amira Haddarah

**Affiliations:** ^1^ Departament d'Enginyeria Agroalimentària i Biotecnologia Universitat Politècnica de Catalunya, BarcelonaTech Castelldefels Spain; ^2^ Doctoral School of Sciences and Technology Lebanese University Hadath Lebanon

**Keywords:** acrylamide, amino acids, potato cultivar, potato models, reducing sugars, sucrose

## Abstract

Amino acids and sugars, along with the thermal processing, are considered the main parameters to control acrylamide formation in fried potatoes. To evaluate which of these parameters had the greatest influence, 10 synthetic potato‐starch‐based models formulated in different amino acid and/or sugar combinations and three potato cultivars were assigned. High‐performance‐liquid chromatography and gas chromatography flame‐ionized‐detectors were applied to quantify amino acids, sugars, and acrylamide. Results showed that reducing sugars and sucrose significantly increased acrylamide formation amongst all potato samples. Synthetic potato models Asn‐GFS contained the highest amount of acrylamide compared to Glu‐Fru and real potatoes (Agria and Kennebec). Thus, sugars were considered critical factors for acrylamide formation in potatoes and remained the most practical way of reducing its production.

## Graphical abstract

The composition of potato cultivars affect acrylamide formation after thermal treatment, therefore synthetic potato models were formulated in order to better understand the effect of sugar and amino acids on acrylamide formation after frying.

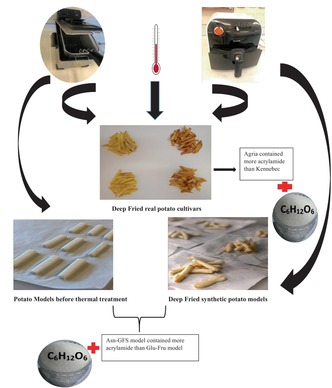

## 1 INTRODUCTION

Acrylamide, formed in thermally heated foods, particularly starchy foods, imposes a major health problem (Bachir et al., 2022). It is formed via the Maillard reaction, a non‐enzymatic browning that happens in foods when amino acids chemically react with carbohydrates, mainly reducing sugars (Zyzak et al., 2003). Acrylamide is classified as a probable carcinogen, and it can cause neurological and reproductive problems (Friedman, 2003; Tareke et al., 2002). French fries, potato chips, and breakfast cereals contain the highest amount of ingested acrylamide and are considered essential contributors to dietary intake across Europe (Muttucumaru et al., 2014; Rydberg et al., 2006). The association between the concentrations of glucose and fructose, asparagine in potatoes, and acrylamide formation during frying is complicated. Some studies concluded that reducing sugar concentration had a major role in acrylamide formation, while others showed that free asparagine concentrations might be the determining factor in the Maillard reaction (Amrein et al., 2003; Becalski et al., 2003; Shepherd et al., 2010). Studies have found that sugars are the limiting factors in the Maillard reaction and are responsible for acrylamide formation in carbohydrate‐rich foods (Amrein et al., 2003, 2004; Knight et al., 2021). For instance, acrylamide production has been examined in model systems containing amino acids and reducing sugars. Correlations have been shown between fructose and acrylamide formation when combined with asparagine (Becalski et al., 2003; Ciesarová et al., 2011; Mottram et al., 2002; Robert et al., 2004; Stadler et al., 2002; Zyzak et al., 2003). However, other studies showed that glucose produced a higher amount of acrylamide when mixed with asparagine (Amrein et al., 2004; Wang & Xu, 2014). Sucrose hydrolysis at high temperatures led to the formation of acrylamide, as demonstrated by (Wolfenden & Yuan, 2008; Yang et al., 2016b). On the other hand, further studies showed that free asparagine can be a major precursor for the Maillard reaction, thus increasing acrylamide formation in food (Mottram et al., 2002). This finding was also supported by many other researchers (Becalski et al., 2003; Oddy et al., 2022; Yasuhara et al., 2003; Yaylayan et al., 2005; Zyzak et al., 2003).

The objectives of the present study were as follows: to formulate 10 synthetic potato‐starch‐based models with different combinations of amino acids and/or sugars for the purpose of better understanding the interaction between acrylamide's precursors; to elucidate whether sugars or amino acids have the greatest impact on acrylamide formation after thermal processing; and to compare the behavior of these simple models with potato tubers on acrylamide formation after frying by investigating the relationship between sugars (glucose, fructose, and sucrose) and amino acids, mainly asparagine and glutamine, among all potato samples. This comparison might help to better understand which precursor had the greatest impact on acrylamide formation in fried potatoes; hence, it might also assist potato industry to control acrylamide content while producing fried potato‐based snacks.

## 2 MATERIALS AND METHODS

### 2.1 Sample

#### 2.1.1 Fresh potato tubers

Three potato cultivars (*Solanum tuberosum* L.) – Agria, Kennebec, and Monalisa – were selected and purchased from Mercabarna (Mercados de Abastecimientos de Barcelona S.A., Barcelona, Spain). The average weight of the potato tubers ranged from 175.09 to 337.60 g. Approximately 6 kg of each potato cultivar of similar size and weight were selected, washed with tap water, and dried on paper towels. Potato tubers were then cut into strips (1 × 1 × 6 cm) with a stainless steel slicer. Sunflower oil was used in the frying. Each cultivar was fried in triplicate under the same frying conditions. After frying, the samples were lyophilized using a Cryodos‐45 freeze‐drying instrument (UPC, Spain), packed in falcon tubes.

#### 2.1.2 Synthetic potato models

According to Amrein et al. (2003), the composition of the synthetic potato models was as follows: potato starch (60%), sugars, amino acids (glutamine and asparagine, 0.25%, 0.6%, respectively), sodium alginate (4.8%), and agar‐agar (1.2%) (responsible for the firmness and elasticity of the models). All ingredients were mixed with distilled water until obtaining a dough‐like product easy to cut using a stainless steel mold. The 10 synthetic potato models were formulated in different combinations of amino acids and/or sugars and named as follows:

1. Prototype model/control model: contained glutamine, asparagine with glucose, fructose, and sucrose.
2. Asn‐GFS/model 1: contained only asparagine with glucose, fructose, and sucrose.
3. Gln‐GFS/model 2: contained only glutamine with glucose, fructose, and sucrose.
4. Glu‐Fru/model 3: contained glutamine, asparagine with glucose, and fructose.
5. Sucrose/model 4: contained glutamine, asparagine with sucrose only.
6. Gln‐Glu/model 5: contained glutamine with glucose only.
7. Gln‐Fru/model 6: contained glutamine with fructose only.
8. Gln‐Suc/model 7: contained glutamine with sucrose only.
9. Asn‐Glu/model 8: contained asparagine with glucose only.
10. Asn‐Fru/model 9: contained asparagine with fructose only.
11. Asn‐Suc/model 10: contained asparagine with sucrose only.

Table S1 summarizes the composition of the 10 synthetic potato models and the types of potato cultivars used in this study before thermal treatment. The weight of the ingredients was expressed g kg^−1^ of dry matter. Samples were divided into triplets and thermally treated using a deep fryer (Mandine) and air‐frying (Tefal). Around 500 g of potato samples were submerged in 5 L of hot sunflower oil and deep‐fried at 170 and 190°C. The % moisture and pH of the models were on average 195–205 g kg^−1^ and 6.2–6.4, respectively. The dry matter and pH of the potato tubers were on average 199–205 g kg^−1^ and 5.89–6.3, respectively (Table 1).

**TABLE 1:** Comparisons of mean acrylamide, acrylamide precursors (amino acids and sugars) after thermal treatment, moisture, and pH by synthetic potato models and potato cultivars.

	Acrylamide (μg kg^−1^)	Glutamine (g kg^−1^)	Asparagine (g kg^−1^)	Fructose (g kg^−1^)	Glucose (g kg^−1^)	Sucrose (g kg^−1^)	Moisture (g kg^−1^)	pH
	Mean ± SD							
Synthetic potato models								
Prototype control model	(—)	2.2 ± 1.6	4.8 ± 1.0	0.2 ± 0.0	0.1 ± 0.0	(—)	192 ± 9.2	6.22 ± 1.6
Asn‐GFS (Model 1)	516.7 ± 29.67	5.2 ± 2.2	(—)	0.1 ± 0	0.4 ± 0.3	0.2 ± 0.0	195 ± 8.9	6.30 ± 1.9
Gln‐GFS (Model 2)	(—)	0.2 ± 0.1	0.7 ± 0.3	0.2 ± 0	0.3 ± 0.1	0.2 ± 0.1	190 ± 9.5	6.29 ± 2.7
Glu‐Fru (Model 3)	438.9 ± 29.78	9.1 ± 1.6	1.0 ± 0.2	0.2 ± 0.1	0.1 ± 0.0	(—)	197 ± 8.5	6.25 ± 1.4
	316.7 ± 94.9	10.0 ± 1.4	(—)	(—)	(—)	(—)	205 ± 7.9	6.27 ± 2.1
Glutamine‐Glucose (Model 5)	(—)	3.7 ± 0.7	0.7 ± 0.4	(—)	(—)	(—)	200 ± 9.1	6.30 ± 1.8
Glutamine‐Fructose (Model 6)	(—)	3.1 ± 0.5	1.0 ± 0.7	(—)	(—)	(—)	191 ± 7.7	6.32 ± 2.3
Glutamine‐Sucrose (Model 7)	(—)	3.0 ± 1.3	0.9 ± 0.5	(—)	(—)	(—)	200 ± 8.8	6.34 ± 2.0
Asparagine‐Glucose (Model 8)	(—)	6.1 ± 4.8	1 ± 0.5	(—)	(—)	(—)	198 ± 8.2	6.38 ± 1.5
Asparagine‐Fructose (Model 9)	(—)	10.1 ± 1.7	0.8 ± 0.5	(—)	(—)	(—)	203 ± 9.3	6.36 ± 1.8
Asparagine‐Sucrose (Model 10)	(—)	2.4 ± 4.5	0.9 ± 0.8	(—)	(—)	(—)	200 ± 5.7	6.40 ± 2.2
Potato cultivars								
Kennebec	338.7 ± 6.5	8.4 ± 8.5	8.1 ± 1.6	(—)	(—)	0.7 ± 0.0	205 ± 7.2	5.89 ± 0.9
Monalisa	205.6 ± 19.4	8.0 ± 3.1	4.7 ± 2.4	1.4 ± 0.1	0.3 ± 0.4	0.7 ± 0.1	202 ± 9.6	6.2 ± 2.1
Agria	441.6 ± 20.8	2.4 ± 2.7	7.8 ± 0.3	0.5 ± 0.8	(—)	1.0 ± 0.1	199 ± 8.3	6.3 ± 1.4

*Note*: Acrylamide limit of quantification (LOQ) using gas chromatography flame‐ionized‐detectors for potato models was 0.5 g kg^−1^. Sugars limit of quantification (LOQ) using high‐performance liquid chromatography is 0.1 g kg^−1^. (—) Means ingredient not present.

### 2.2 Determination of amino acids: asparagine and glutamine

Asparagine and glutamine were quantified using the kit (K‐ASNAM) of Megazyme international 2014 (Yang et al., 2016b). Two grams of the homogenized lyophilized fried sample were mixed with 60 mL of distilled water and incubated in the oven at 60°C for 5 min, then filled with distilled water until it reached 100 mL. The solution was filtered using Whatman 1 filter paper. Then 0.1 mL of the filtrate was taken for analysis. Samples were analyzed in triplicate.

For the kit analysis (discussed in detail in our previous research; data not shown): 0.1 mL of the extract was mixed with a series of enzymes, and then the absorbance A_1_ was read at *ʎ* = 340 nm. Then the reaction continued, and absorbance A_2_ was read at *ʎ* = 340 nm. The glutamine concentration was calculated as follows: Concentration of glutamine (g kg^−1^ DM) = [(A_1_ − A_2_)_sample_ − (A_1_ − A_2_)_blank_] × 0.5427.

To calculate asparagine concentration, the reaction continues after adding other enzymes, and absorbance A3 was read at *ʎ* = 340 nm. Concentration of asparagine (g kg^−1^ DM) = [(A_2_ − A_3_)_sample_ − (A_2_ − A_3_)_blank_] × 0.4949. Ammonia absorbance was obtained as follows: Δ ammonia = (A_1_ − A_2_)_ammonia sample_ − (A_1_ − A_2_)_ammonia blank_. To calculate ammonia concentration: [(A_1_ − A_2_)_ammonia sample_ − (A_1_ − A_2_)_blank_] × 0.06325. Each sample was analyzed in triplicate.

### 2.3 Quantification of acrylamide by the GC‐FID method

According to Yang et al. (2016b) procedure, with some modifications, the concentration of acrylamide was determined. Three grams of the lyophilized fried samples were mixed with 20 mL of a 0.1% formic acid solution and centrifuged (1507 *g* for 10 min at 24°C). Then, a 3 mL aliquot of the clarified aqueous was passed through the SPE tube. The acrylamide residue in the SPE was eluted with 2 mL of acetone using gravity flow and collected for analysis. The GC analysis was performed on an Auto‐System GC equipped with a flame ionization detector (FID) (Hewlett Packard 5890 series II) following the procedure by Sun et al. (2012). The column used was an Agilent HP‐FFAP capillary (length = 25 m, i.d. = 0.2 mm, and thickness = 0.3 μm), and the analysis conditions were as follows: the initial column temperature was settled at 100°C for 0.5 min, then raised at a gradient of 10°C per minute to 200°C; the temperatures of the injector and detector were set to 250 and 260°C, respectively; helium was used as the carrier gas at a flow rate of 1 mL min^−1^ and a splitless of 1 min; and the injection volume was 1 μm. The quantification limit of the proposed method for potato samples was 0.5 g kg^−1^. The results were expressed as μg_acrylamide_ kg^−1^ of sample (DW). All samples were done in triplicate.

### 2.4 Determination of sugars (glucose, fructose, and sucrose) by HPLC

Ten mL of 80% ethanol with 5 g of lyophilized fried samples were agitated for 15 min. Then, the samples were centrifuged at 1200 *g* for 10 min. The extraction was performed three times. First, the supernatant was collected and put aside. Then, the process was repeated twice, and a new supernatant was added to the previous one. The extract was concentrated at 65°C for ethanol evaporation until reaching a total volume of 10 mL. For HPLC analysis, 3 mL of the extract was filtered (0.45 nm pore‐size), and then 20 μL of each filtrate was injected into a Hewlett‐Packard series 1100 injector with a Beckman 156 Refraction Index Detector. The separation was performed using a Tracer carbohydrate column (5 μm, 250 × 4.6 mm) (Teknoroma). The mobile phase consisted of acetonitrile/water (75:25, v/v), and the flow rate was 1.4 mL min^−1^. Individual sugars were identified and quantified using external standards. Each sample was analyzed in triplicate. The sugar contents were expressed as g kg^−1^ of sample DM (Yang et al., 2016a).

### 2.5 Statistics

Data were entered and analyzed using Statistical Package for Social Science (SPSS) version 24. Continuous variables were presented as means and standard deviations (STDEV). Pearson correlation and scatter plots were conducted to examine the relation between acrylamide concentration and the independent variables (temperature, technique, synthetic models, and potato tubers). Simple and multiple linear regression analyses were used to study the associations between acrylamide concentration and oil uptake and acrylamide precursors, sugars, on the proposed models and potato tubers, adjusting for temperature and technique. Simple and multiple logistic regression analysis was performed to examine the likelihood of exceeding the threshold of acrylamide (below vs. above threshold) for acrylamide precursors and sugars in the proposed models and potato tubers, adjusting for temperature and technique. Results from the linear regression models were expressed as Beta coefficients (*β*) with 95% CI. Results from the logistic regression models were expressed as odds ratios (OR) with a 95% CI. All reported *p*‐values were based on two‐sided tests and were compared with a significance level of 5%.

## 3 RESULTS AND DISCUSSION

### 3.1 Comparison of acrylamide formation in synthetic potato models and potato tubers

#### 3.1.1 Effect of sample composition on acrylamide content

Table 1 represents a summary of the mean values of acrylamide and acrylamide precursors (amino acids and sugars) after thermal treatment in synthetic potato models and potato cultivars. In synthetic potato models, the prototype model contained a small amount of acrylamide after frying. With regard to models, the concentration of acrylamide was found to be the highest in the Asn‐GFS model (516.7 μg kg^−1^) > Glu‐Fru model (438.9 μg kg^−1^) > Sucrose model (316.7 μg kg^−1^) (borderline). The aim of the present study was concentrated mainly on the potato samples that produced high amounts of acrylamide exceeding the LOQ after thermal treatment.

##### Synthetics models

The concentration of asparagine in the synthetic potato models after frying ranged from 0.0 to 4.8 g kg^−1^, whereas the concentration of glutamine in the models after frying ranged from 0.2 to 10.1 g kg^−1^. In glutamine‐based models (5, 6, and 7), acrylamide was not detected, and that could be due to two reasons: (1) glutamine is not a major contributor to the Maillard reaction; (2) sugars in these models were added separately with glutamine; hence confirming that sugar, when put alone, cannot react with glutamine to form acrylamide. Moreover, even when the three sugars were added together with glutamine, as in Gln‐GFS (model 2), acrylamide was likewise not detected and formed. Hence, glutamine‐based models might additionally prove that glutamine is not a key contributor to acrylamide development. Our finding was also supported by Amrein et al. (2003) and Champrasert et al. (2022). On the other hand, in asparagine‐based models (8, 9, and 10), when asparagine was added with only one sugar, acrylamide was not formed. However, in Asn‐GFS (model 1), when asparagine was mixed with glucose, fructose, and sucrose altogether, a huge amount of acrylamide (516.7 μg kg^−1^) was detected using GC‐FID. This finding was also proved by Robert et al. (2004) and Knol et al. (2005, 2010). In Asn‐GFS (model 1), presence of sucrose along with the reducing sugars might have increased acrylamide formation due to its hydrolysis (Amrein et al., 2004; Wang & Xu, 2014; Wolfenden & Yuan, 2008; Yang et al., 2016b). It is worth mentioning that when formulating glutamine‐based models and asparagine‐based models, glutamine was not added to asparagine‐based models, and vice versa. However, after frying the synthetic potato strips, asparagine was detected in glutamine‐based models, and glutamine was detected in asparagine‐based models. This can be explained by the theory that there is an inter‐conversion between glutamine and asparagine during the thermal treatment. In general, glutamine at high temperatures degrades and releases ammonium (source of the amine group) and, at the same time, promotes the oxidative deamination of glutamate, which results in the formation of α‐ketoglutarate that forms the backbone of Asparagine. Another proposed pathway for the formation of the asparagine backbone is from the intermediates of the glycolytic route (Puigserver, 2018). Nevertheless, since the potato models are synthetic and the reactions of the quantification of amino acids are performed in‐vitro the proposed explanation of the inter‐conversion between asparagine (C_4_H_8_N_2_O_3_) and glutamine (C_5_H_10_N_2_O_3_) within the same model might be due to methylation/de‐methylation. This reaction is mediated by the glutamate dehydrogenase enzyme added during the experiment, where NADPH is converted to NADP^+^ and CO_2_ is released, and the CH_3_ group is donated from glutamine to asparagine and vice versa (Bachir et al., 2023).

##### Potato cultivars

In potato cultivars, Agria produced a higher acrylamide concentration (441.6 μg kg^−1^) > Kennebec (338.7 μg kg^−1^) > Monalisa (205.6 μg kg^−1^). In real potatoes, asparagine concentration ranged from 4.7 to 8.1 g kg^−1^. As for glutamine concentration, it ranged from 2.4 to 8.4 g kg^−1^. The sugar concentration in all potato samples decreased after frying, which proved their interaction and participation in the Maillard reaction.

### 3.2 Effect of thermal processing on amino acids and sugar content in synthetic potato models and potato cultivars

After thermal treatment, acrylamide was only formed in the following samples (Agria, Kennebec, Monalisa, Asn‐GFS, Glu‐Fru, and sucrose models). Therefore, the analysis was conducted on the previously mentioned samples in order to investigate the behavior of amino acids and sugars on acrylamide formation. In potato cultivars, acrylamide was produced in high amounts in Agria and Kennebec, and in borderline amounts in Monalisa. When studying the percentage of reacted acrylamide's precursors, as shown in Table 3, sucrose and asparagine were found to be the main reactants leading to acrylamide formation. In Agria, 20% of reacted sucrose, 71.1% of reacted asparagine, and 89.5% of reacted glutamine yielded 441.6 μg kg^−1^of acrylamide. Furthermore, in Kennebec, 32% of reacted sucrose and 88% of reacted asparagine yielded 338.7 μg kg^−1^ of acrylamide (Table 2). The higher amount of acrylamide produced in Agria as compared to Kennebec might be explained by the synergistic effect between glutamine and asparagine on acrylamide formation, and that was also elaborated in another study (Devleeschouwer et al., 2009). On the other hand, in Monalisa, when all acrylamide's precursors reacted in different percentages among each other, acrylamide was the least formed 205.6 μg kg^−1^. Hence, in potato cultivars, sucrose and asparagine seemed to play a major role in acrylamide formation. With respect to synthetic potato models, reducing sugars showed to have a main impact on acrylamide formation. This finding was also supported in a previous study (Wang & Xu, 2014). For instance, when all the reducing sugars reacted with all the asparagine in the models, explaining their total participation in the Maillard reaction and, as a result, favoring the formation of Maillard's byproducts. Hence, glucose, fructose, and asparagine appeared to significantly increase acrylamide formation. In the sucrose model, when sucrose reacted 100% with glutamine 74% and asparagine, this led to the formation of acrylamide, but in a smaller amount as compared to the Asn‐GFS and Glu‐Fru models (Table 2). Therefore, these findings confirm that reducing sugars, sucrose, and asparagine were the major contributors in the Maillard reaction, thus leading to acrylamide formation.

**TABLE 2:** Percentage of reactant acrylamide's precursors after thermal processing.

Potato samples	% Reactant sucrose	% Reactant fructose	% Reactant glucose	% Reactant glutamine	% Reactant asparagine
Potato cultivars					
Agria	20	25.23	0	89.46	71.12
Monalisa	41.7	73.43	29.51	90.94	94.43
Kennebec	32	0	0	0	88.17
Potato models					
Sucrose model	100	0	0	73.92	100
Glu‐Fru	0	100	100	73.25	100
Asn‐GFS	83.2	100	100	18.52	100

*Note*: Percentage calculated = ([precursor] after thermal treatment − [precursor] before thermal treatment/[precursor] after thermal treatment × 100).

### 3.3 Relationship between acrylamide concentration in potato samples, amino acids, and sugars

Simple linear regression of acrylamide in all potato samples showed that Asn‐GFS (model 1) and Glu‐Fru (model 3) synthetic potato models significantly increased acrylamide formation (*β* = 390.2 (182.5–597.93), *p* < .0001; *β* = 305.66 (86.32, 524.99), *p* = .007), respectively (Table 3). In multiple linear regressions, these synthetic potato models remained statistically significant (*p* < .001). In addition, the sucrose synthetic model showed borderline formation in acrylamide (*β* = 290.93 (152.10, 429.75), *p* < .001). It is important to note that in the sucrose model, sugars disappeared after frying, which proved their participation in Maillard reaction, and hence acrylamide was detected at the borderline. With respect to real potatoes, simple linear regression showed that Agria potatoes were associated with acrylamide formation (*β* = 295.76 (−20.34, 611.86), *p* = .06, borderline significance). Results from multiple logistic regression showed that both Agria and Kennebec potato species produced a higher amount of acrylamide compared to other models (*β* = 415.87 (225.08, 606.670), *p* < .001 and *β* = 321.98 (152.10, 429.75), *p* < .001) (Table 3). It is worth mentioning that the amount of sugar decreased as the acrylamide concentration increased.

**TABLE 3:** Simple and multiple linear regression of associations between acrylamide concentration and potato samples of different compositions (*n* = 50).

	Simple linear regression	Multiple linear regression
	Acrylamide concentration (μg kg^−1^)	Acrylamide concentration (μg kg^−1^)
	*β* (95% CI)	*β* (95% CI)
Potato models		
Asn‐GFS (model 1)	**390.2 (182.5–597.93), *p* < .0001**	**490.93 (−20.54, 72.01), *p* < .001**
Glu‐Fru (model 3)	**305.66 (86.32, 524.99), *p* = .007**	**413.15 (274.33, 551.97), *p* < .001**
Sucrose (model 4)	172.80 (−58.41, 404.02), *p* = .14	**290.93 (152.10, 429.75), *p* < .001**
Potato cultivars		
Agria	295.76 (−20.34, 611.86), *p* = .06	**415.87 (225.08, 606.670), *p* < .001**
Kennebec	188.60 (−134.36, 511.52), *p* = .25	**321.98 (152.10, 429.75), *p* < .001**
Monalisa	49.87 (−277.36, 377.09), *p* = .76	179.81 (−10.97, 370.61), *p* = .064
Temperature		
170°C	98 (−118, −137), *p* = .88	90 (−95, −113), *p* = .26
190°C	0	0
Technique		
Air frying	−76 (−212, −59), *p* = .26	−57 (−168, −54), *p* = .31
Deep frying	0	0

*Note*: *β* of the dependent variable acrylamide concentration is presented with 95% CI using simple and multiple linear regression. Significance at *p* < .05. 190°C and deep‐frying cooking techniques were taken as references in the statistical analysis.

The highest amount of acrylamide was formed in the synthetic potato models in the following order: Asn‐GFS > Glu‐Fru > Sucrose. This finding supported the significant role of sugars as major precursors in acrylamide formation, as likewise demonstrated by Amrein et al. (2003) and Henao Toro et al. (2022). In line with our finding, Parker et al. (2012) showed that the ratio of fructose:glucose had a great impact on acrylamide formation in French fries. With respect to potato cultivars, acrylamide concentration was found to be highest in Agria > Kennebec > Monalisa. This result is in line with Yang et al.'s (2016b) finding. The low concentration of acrylamide in Monalisa cultivar could be due to its low content of sugars, mainly fructose, as compared to Agria and Kennebec. For instance, low sugar levels limit the formation of acrylamide and favor the competition of asparagine with other amino acids (Knight et al., 2021). It is also worth stating that acrylamide formation in potato tubers was found to be cultivar‐specific where the concentration of acrylamide varied (Dite Hunjek et al., 2021; Liyanage et al., 2021). Moreover, results showed that temperature did not have any statistical significance on acrylamide formation in the studied samples, contrary to other studies findings (Palazoglu et al., 2010; Yang et al., 2016b). Furthermore, air frying showed a decrease in acrylamide in the potato samples, which was in line with previous findings (Haddarah et al., 2021; Lee et al., 2020).

As such, we illustrate the relationship between our main dependent variable (acrylamide concentration) and our independent variables (potato samples, amino acids, and sugars) in linear regression Equation 1:

(1) y=ax1+bx2+cx3+dx4+ex5+fx6+constant

where *y* axis denotes the acrylamide concentration, *x* axis refers to the type of potato model, and *a*–*f* represent the beta coefficients (i.e. rate of change).

The obtained parameter estimates for the linear regression are presented in Equation 2:

(2) $$\begin{array}{c} \text{Acrylamide concentration}\left(\frac{\mathrm{\mu g}}{\mathrm{kg}}\right)=\mathrm{Asn}‐\mathrm{GFS}\;\left(491\right)+\mathrm{Glu}‐\mathrm{Fru}\;\left(413\right)\\ \;+\text{Sucrose}\;\left(290\right)+\text{Agria}\;\left(416\right)+\text{Kennebec}\;\left(322\right)\\ \;+\text{Monalisa}\;\left(180\right)+26 \end{array}$$

### 3.4 Rate of change in amino acids and sugars on acrylamide formation

#### 3.4.1 Effect of precursors on acrylamide content

**FIGURE 1:**
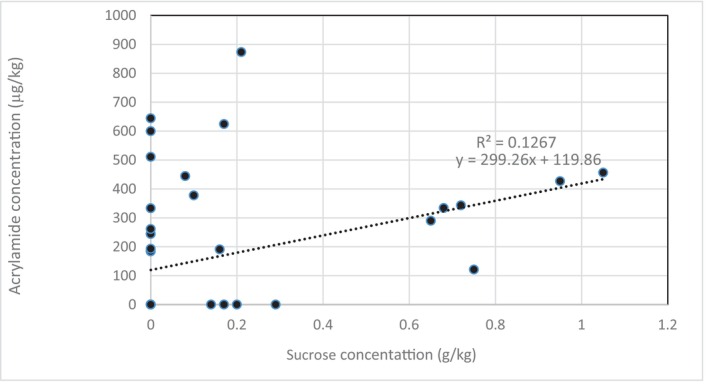
Scatter plot of correlation between acrylamide and sucrose concentration in all potato samples. The dotted line presents the trend line showing a positive and significant association between acrylamide and sucrose, as depicted by the following equation: *y* = 299.26*x* + 119.86; *R* ^2^ change = 12.67%. This means that sucrose explains about 13% of the change in acrylamide concentration.

When looking at amino acids and sugars separately, we found that fructose increased borderline acrylamide formation in all the samples (*r* = .23, *p* = .05) (Table 4). This result was in line with previous studies, which showed that reducing sugars react to produce high levels of acrylamide (Ciesarová et al., 2011; Gökmen & Şenyuva, 2006; Surdyk et al., 2004). Hence, through the Amadori rearrangement, aldose is transformed into a ketose sugar derivative. It is supposed that in the reaction between ketoses (fructose) and amino groups, ketosylamines are formed, followed by Heyns rearrangement to form 2‐amino‐2‐deoxyaldoses. Sucrose increased acrylamide significantly, where acrylamide content may be obtained from the hydrolysis of sucrose (*r* = .36, *p* = .06) (Table 5) (Amrein et al., 2004; Halford et al., 2012; Henao Toro et al., 2022; Orsák et al., 2022; Yang et al., 2016b). Figure 1 represents a scatter plot of sucrose concentration versus acrylamide concentration for all potato models. The figure depicts a positive correlation between sucrose and acrylamide (*y* = 299.26*x* + 119.86, *R* ^2^ = .1267). This means that sucrose explained about 13% of the change in acrylamide concentration. A bivariate correlation was conducted between the mean concentrations of sucrose and acrylamide. Results showed that there is a weak, positive, and significant correlation between sucrose and acrylamide (Pearson correlation = .356, *p* = .01). This means that as the concentration of sucrose increases, the concentration of acrylamide increases. In Figure 1, the scatter plot showed a weak nonlinear correlation between the two variables, and the data points were scattered. Based on the spearman correlation, which is more robust than pearson, that is, more sensitive to nonlinear relationships, we found that the Spearman *ρ* = .5 and *p*‐value < .01 is significant, meaning that there is a moderate, positive, significant association. However, there are some data points that show that even at lower levels of sucrose, a high concentration of acrylamide was found. Few data points were aligned on the linear trend line (*y* = 299.26*x* + 119.86, *R* ^2^ = 12.67%). Moreover, in adjusted models, only sucrose was found to significantly have an impact on acrylamide (*β* = 371.02 (4.89, 737.14), *p* = .047). For every one unit (g kg^−1^) increase in sucrose at the end of the frying process, there is an increase in acrylamide concentration by 371 μg kg^−1^, *p* = .047. It is worth saying that from the present study, it was concluded that the impact of sugars on acrylamide formation is much more significant than amino acids (asparagine and glutamine). This finding is in line with previous research (Amrein et al., 2003, 2004; Knight et al., 2021; Yang et al., 2016b).

**TABLE 4:** Pearson correlations and linear regression analyses of associations between acrylamide concentration and acrylamide precursors in all potato samples.

Parameters	Pearson correlation	*p*‐value	Acrylamide concentration	Acrylamide concentration
			Simple regression (μg kg^−1^)	Multiple regression (μg kg^−1^)
			*β* (95% CI)	*β* (95% CI)
Sugars				
Glucose	.14	.16	189.73 (−192.34, 570.61), *p* = .32	7.49 (−426.82, 441.81), *p* = .97
Fructose	.23	**.05**	169.14 (−34.30, 372.58), *p* = .1	66.86 (−272.13, 405.85), *p* = .69
Sucrose	.36	.06	**299.26 (71.23, 527.30), *p* = .01**	**371.02 (4.89, 737.14), *p* = .047**
Amino acids				
Glutamine	.13	.18	5.73 (−6.25, 16.78), *p* = .36	4.98 (−6.60, 16.55), *p* = .39
Asparagine	.20	.80	14.29 (−5.80, 34.37), *p* = .16	−15.86 (−53.09, 22.18), *p* = .40

*Note*: *β* of the dependent variable acrylamide concentration is presented with 95% CI using simple and multiple linear regression. Significance at *p* < .05.

**TABLE 5:** Multiple logistic regression of the likelihood of exceeding the acrylamide concentration in different potato samples.

	Acrylamide concentration (μg kg^−1^)
	Odds ratios (95% CI), *p*‐value
Potato models	
Asn‐GFS (model 1)	**10.94 (1.07, 120.43), *p* = .05**
Glu‐Fru (model 3)	**10.80 (1.01, 115.43), *p* = .05**
Sucrose (model 4)	3.20 (0.40, 25.31), *p* = .27
Potato cultivars	
Agria	0.82 (0.09, 7.02), *p* = .85
Kennebec	0.82 (0.09, 7.02), *p* = .85
Monalisa	0.22 (0.02, 2.53), *p* = .23

*Note*: Significance at *p* < .05.

### 3.5 Study the odds ratios of acrylamide content in potato models

#### 3.5.1 Sample composition effects

A new categorical dichotomous variable was created based on the cut‐off of the acrylamide level for each type of potato model, where “0” indicates below the threshold level and “1” indicates above the threshold level. Multiple logistic regression results showed that Asn‐GFS (model 1) and Gluc‐Fru (model 3) synthetic potato models increased almost 11 times the risk of exceeding the acrylamide threshold level (OR = 10.80, 95% CI: 1.01, 115.43, *p* = .05) (Table 5). These conclusions concurred that the composition of the potato model had a major impact on acrylamide formation. Hence, sugar content played a major role in producing acrylamide along with amino acids, mainly asparagine and glutamine. It is important to note that potato cultivars produced acrylamide after frying; however, mean acrylamide concentration was lower than the content of acrylamide formed in the synthetic models. Results showed that potato cultivars reduce the likelihood of exceeding acrylamide. Kennebec and Agria decreased the odds by 20% (OR = 0.82, 95% CI: 0.095, 7.02, *p* = .85). Monalisa was shown to greatly minimize the risk of exceeding acrylamide content (OR = 0.22, 95% CI: 0.02, 2.53, *p* = .23). This may be explained by the fact that real potatoes contain several phytochemicals, such as flavonoids and carotenoids, known as bioactive compounds, that are beneficial to human health. These phytochemicals might act as protective factors and decrease acrylamide formation in potato pulp (71 μg acrylamide per kg dry matter) (Trabert et al., 2022). Another interpretation could be due to the low content of sugars that are initially present in Monalisa cultivars (Knight et al., 2021; Yang et al., 2016b).

## 4 CONCLUSION

The present comparative study demonstrated that acrylamide content was affected mainly by the composition of sugars rather than amino acids, where glucose, fructose, and sucrose can be considered as main contributors in the Maillard reaction. Moreover, sucrose showed a major impact on acrylamide formation in all the samples (synthetic models and potato cultivars). Consequently, sugars were considered key and critical factors for acrylamide formation in potatoes and remained the most practical way of reducing its production in potato products. Thus, this might be achieved either by optimizing cultivars or controlling the storage conditions of potatoes. Moreover, the mitigation of acrylamide in potatoes can also be achieved by some of the traditional techniques, such as soaking, blanching, and adding natural herbs to the oil. This comparison might help the potato industry to control acrylamide content while producing fried potato snacks.

## AUTHOR CONTRIBUTIONS

**Nivine Bachir:** Conceptualization (equal); data curation (equal); formal analysis (equal); investigation (equal); methodology (equal); software (equal); visualization (equal); writing – original draft (equal). **Hadiya Akkoum:** Data curation (equal). **Montserrat Pujola:** Conceptualization (equal); data curation (equal); investigation (equal); methodology (equal); supervision (equal); visualization (equal). **Franscesc Sepulcre:** Supervision (equal); visualization (equal). **Amira Haddarah:** Conceptualization (equal); methodology (equal); supervision (equal); visualization (equal).

## FUNDING INFORMATION

Erasmus K^+^ and Lebanese University (LU).

## CONFLICT OF INTEREST STATEMENT

The authors declare no conflicts of interest.

## DECLARATION

This paper has not been published before. It is not under consideration for publication elsewhere. All authors approved its submission to the *Food Science & Nutrition Journal*.

## Supporting information


Table S1

## Data Availability

Research data are not shared.
