# Supplementary material for: Impact of amino acids and sugars after thermal processing on acrylamide formation in synthetic potato models and real potatoes
Source: Food Sci Nutr. 2023 Nov 15;12(2):1046–55. doi: 10.1002/fsn3.3818 (PMC10867486; doi:10.1002/fsn3.3818)
Supplement: Supplementary file 1 — Table S1 [file FSN3-12-1046-s001.docx]

| **Table S 1: Composition of synthetic potato models and potato cultivars before thermal treatment: Weight of ingredients (g.Kg^-1^ DM) in the studied samples.** | | | | | | | | | | | | | | |
| --- | --- | --- | --- | --- | --- | --- | --- | --- | --- | --- | --- | --- | --- | --- |
| **Raw weight**  (g.Kg^-1^ DM) | **Glutamine Models (Gln)** | | | **Asparagine Models (Asn)** | | | **(Gln/Asn)**  **Prototype Model** | **Glu/Fru**  **Model**  **3** | **Sucrose**  **Model**  **4** | **Asn/GFS**  **Model**  **1** | **Gln/GFS**  **Model**  **2** | **Potato cultivars** | | |
|  | **(Gln-Glu)**  **Model 5** | **(Gln-Fruc)**  **Model 6** | **(Gln-Sucr)**  **Model 7** | **(Asn-Glu)**  **Model 8** | **(Asn-Fruc)**  **Model 9** | **(Asn-Sucr)**  **Model 10** |  |  |  |  |  | **Agria** | **Kennebec** | **Monalisa** |
| Glutamine | 2.5 | 2.5 | 2.5 | 0 | 0 | 0 | 2.5 | 2.5 | 2.5 | 0 | 2.5 | 0.5 | 0.43 | 0.6 |
| Asparagine | 0 | 0 | 0 | 5.7 | 5.7 | 5.7 | 5.7 | 5.7 | 5.7 | 5.7 | 0 | 2.25 | 0.93 | 0.8 |
| Glucose | 1.2 | 0 | 0 | 1.2 | 0 | 0 | 1.2 | 1.2 | 0 | 1.2 | 1.2 | 1.2 | 4.6 | 0.43 |
| Sucrose | 0 | 0 | 1.8 | 0 | 0 | 1.8 | 1.8 | 0 | 1.8 | 1.8 | 1.8 | 1.25 | 1.03 | 1.2 |
| Fructose | 0 | 0.7 | 0 | 0 | 0.7 | 0 | 0.7 | 0.7 | 0 | 0.7 | 0.7 | 0.8 | 2.53 | 0.37 |
| Agar | 18.3 | 18.76 | 18.94 | 18.51 | 17 | 18 | 17.1 | 17.1 | 17.1 | 17.1 | 17.1 | (--) | (--) | (--) |
| Water | 655 | 641.7 | 663.1 | 715 | 765.6 | 669.5 | 707.3 | 700 | 700.2 | 701.1 | 702.1 | (--) | (--) | (--) |

Note: (--) means not present
